# Editorial: Mechanism and treatment for pancreatic cancer metastases

**DOI:** 10.3389/fonc.2024.1424817

**Published:** 2024-05-14

**Authors:** Wenyi Guo, Dong Wu, Jianwei Xu, Lei Wang

**Affiliations:** ^1^ Division of Pancreatic Surgery, Department of General Surgery, Qilu Hospital of Shandong University, Jinan, China; ^2^ Department of Systems Biology, Beckman Research Institute of City of Hope, Monrovia, CA, United States

**Keywords:** pancreatic cancer, metastases, chemotherapy, immunotherapy, tumor immune microenvironment (TIME)

Over half of the patients diagnosed with pancreatic cancer present with distant metastases at the time of their initial diagnosis (1). In such cases, surgical interventions fail to enhance survival rates for those suffering from metastatic pancreatic ductal adenocarcinoma (mPDAC). Therefore, alternative therapeutic strategies, including chemotherapy, immunotherapy, and localized treatments, are imperative to consider at this stage.

Several studies have demonstrated that the FOLFIRINOX regimen or the combination of albumin-bound paclitaxel and gemcitabine surpasses the efficacy of gemcitabine monotherapy in managing mPDAC (2, 3), leading to improvements in patient survival quality (4). Furthermore, immunotherapy targeting PD-1, in conjunction with AG regimens, has also been shown to significantly improve survival outcomes (5). Additionally, nanoliposomal irinotecan has emerged as another promising option offering survival benefits to mPDAC patients (6). Transarterial chemoembolization (TACE) also stands out as an effective treatment for liver metastasis in pancreatic cancer (7). Despite these encouraging advancements, mPDAC continues to be a formidable challenge, necessitating ongoing research to refine therapeutic strategies.

Non-coding RNAs are crucial in the progression of various cancers, including PDAC and its metastases (8). Research into ncRNAs can facilitate early detection, prognosis, and the creation of treatment plans for mPDAC patients. Jafari et al. conducted a review focusing on long non-coding RNAs (LncRNAs) and microRNAs (miRNAs) implicated in mPDAC, underscoring their potential therapeutic significance.

Immunotherapy has become a pivotal treatment modality for various solid tumors in the last decade. However, the efficacy of immunotherapy in PDAC is significantly hindered by its low mutation rate, weak immunogenicity, and inadequate T cell activation (9, 10). Gao et al. discovered that liver metastases exhibit a more immunosuppressive microenvironment than primary tumors. Their study also demonstrated that the modified FOLFIRINOX (mFOLFIRINOX) regimen significantly enhances the tumor immune microenvironment (TiME) by promoting CD8+ T cell infiltration and reducing regulatory T cells (Tregs). Conversely, gemcitabine-based chemotherapy fails to ameliorate TiME. Furthermore, Sun et al. developed a TiME score based on the immune-related risk profile gene RARRES3, which strongly correlates with the effectiveness of immunotherapy in pancreatic cancer. This score offers predictive and prognostic insights and suggests new immunotherapeutic approaches for patients with this condition.

Pancreatic cancer is frequently associated with perineural invasion (PNI), a key factor contributing to the intense pain experienced by patients (11). The interaction between pancreatic cancer cells and peripheral nerves not only triggers neurogenesis but also promotes the proliferation of the cancer cells. Alterations in the nerve plexus within the pancreatic head are strongly associated with early postoperative liver metastasis and heightened mortality rates, leading to an unfavorable prognosis (11, 12). Wang et al. explored the intricate interactions among neural cells, tumor cells, and stromal cells. Their discussion highlights the potential of therapies that target neural pathways for therapeutic benefits and pain relief in treating periampullary carcinoma.

Liver metastases are the most common in pancreatic cancer, yet detecting small asymptomatic metastases proves challenging due to their low detection rate. Brain metastases, in particular, are often elusive (13). In their study on pancreatic cancer brain metastases (BM), Gouton et al. detailed the clinical characteristics associated with these cases. The authors suggest that surgical intervention could enhance survival for patients with brain metastases, underscoring the potential therapeutic advantages of surgical treatment for BM in pancreatic cancer.

In addition to PDAC, other histologic types of pancreatic cancer exist, including pancreatic squamous cell carcinoma (PSCC), which often shows metastasis at initial diagnosis (14). Ford et al. analyzed the clinical features of PSCC using the SEER database, highlighting the potential benefits of surgical intervention in oligometastatic cases for appropriate candidates. The study calls for further research with larger cohorts to explore the impact of treatment modalities on survival rates in this understudied malignancy.

While managing metastatic pancreatic adenocarcinoma remains challenging, the presence of diverse treatment options offers hope. Moreover, ongoing basic research is actively identifying novel drugs and agents for mPDAC treatment, indicating the need to initiate clinical trials to address this ailment. Overall, this topic introduces fresh and compelling perspectives on mPDAC, with the aim of advancing its treatment strategies.

## Author contributions

LW: Writing – review & editing, Supervision, Formal Analysis, Conceptualization. WG: Writing – original draft, Validation, Methodology, Investigation. DW: Writing – review & editing, Supervision, Formal Analysis. JX: Writing – review & editing, Methodology.
